# Ion-Imprinted Chitosan-Based Localized Surface Plasmon Resonance Sensor for Ni^2+^ Detection

**DOI:** 10.3390/s22229005

**Published:** 2022-11-21

**Authors:** Xiujuan Zhong, Li Ma, Guolu Yin

**Affiliations:** 1 College of Chemistry & Chemical Engineering, Chongqing University, Chongqing 400044, China; 2 Key Laboratory of Optoelectronic Technology & Systems (Ministry of Education), Chongqing University, Chongqing 400044, China

**Keywords:** optical fiber sensor, nickel ions sensing, localized surface plasmon resonance, ion-imprinted technique, dip coating technique

## Abstract

Heavy metals are important sources of environmental pollution and cause disease in organisms throughout the food chain. A localized surface plasmon resonance sensor was proposed and demonstrated to realize Ni^2+^ detection by using ion-imprinted chitosan. Au nanoparticles were coated on the multimode fiber to excite the local surface plasmon resonance, and Ni^2+^-imprinted chitosan was then functionalized by using the dip coating technique. Ethylene diamine tetra-acetic acid was used to release the Ni^2+^ ions and hence form countless voids. Ni^2+^ was refilled into the voids to increase the refractive index of the sensing material, thus realizing the measurement of Ni^2+^ by monitoring the wavelength shift in the localized surface plasmon resonant peak. The coating thickness of the Ni^2+^–chitosan gel was optimized to obtain greater sensitivity. Experimental results show that the proposed Ni^2+^ sensor has a sensitivity of 185 pm/μM, and the limit of detection is 0.512 μM. The comparison experiments indicated that the ion-imprinted chitosan has better selectivity than pure chitosan.

## 1. Introduction

Heavy metal pollution has been causing great harm to the environment and organisms. Nickel ions (Ni^2+^) are an essential element for the human body, and their content in the human body is very low. However, nickel can cause skin allergies, cardiovascular disease, and respiratory disease once its concentration exceeds a certain upper limit [1,2,3]. At present, many technologies have been developed for detecting metal ions, for example, visible spectroscopy [4], inductively coupled plasma-mass spectrometry [5], and atomic absorption spectrometry [6,7]. However, these detection technologies require expensive equipment, complex operation, and professional operators. The detection of metal ions can also be realized by monitoring the change in the refractive index. Surface plasmons are extremely sensitive to the changes in the refractive index of the surrounding medium and have thus been widely explored for sensing applications [8,9]. On the other hand, optical fiber sensing technologies have been attracting great attention due to their unique advantages of miniaturization, high sensitivity, easy integration, suitability for remote sensing, etc. Among them, localized surface plasmon resonance (LSPR) sensors are commonly used for metal detection [10,11,12]. LSPR generates the surface plasmon modes, and the electromagnetic field remains localized in a nanoscale region around the nanoparticle–dielectric interface [13,14,15,16,17]. The sensing material is usually coated on the optical fiber, and the presence of Ni^2+^ is detected by changing the refractive index surrounding the optical fiber, which results in a change in the LPSR spectrum.

Optical fiber modification is one of the key factors for LSPR-based heavy metal sensors. The layer-by-layer method is used to deposit thin films on the optical fibers by using oppositely charged polyelectrolytes. M. E. Martínez-Hernández et al. built up a polyelectrolyte structure by using poly(allylamine hydrochloride) and poly(acrylic acid) as the positively and negatively charged polyelectrolytes [18], and their method realized the measurement of Hg^2+^ with a 0.1 ppm limit of detection. Chitosan (CS) is another polyelectrolyte used for modifying optical fibers because of its rich hydroxyl and amino groups [19,20,21,22,23,24]. For example, Z. Chen et al. proposed a surface plasmon resonance (SPR) sensor for Hg^2+^ detection by coating an optical fiber with chitosan and polyacrylic acid using the layer-by-layer method [25]. However, CS can absorb a variety of metal ions, leading to cross-sensitivity problems. Ion imprinting technology is used to form a special binding site for metal ions in polymer materials. The method embeds metal ions through the cross-linking between metal ions and polymer materials and then takes the metal ions out of the insertion sites in a suitable way. Ion imprinting is an extension of molecular imprinting, in which target ions are used as templates to prepare ion-imprinted polymers with specific ion recognition. Therefore, ion imprinting makes the polymer material highly specific for template metal ions [26,27].

In this paper, we proposed a Ni^2+^ sensor based on LSPR by using the ion printing technique. Gold nanoparticles (AuNPs) were firstly deposited on the surface of a multimode fiber, which was then coated with Ni^2+^-templated CS gel using the dip coating technique. Finally, an EDTA solution was applied to release the Ni^2+^ ions and generate specific voids for Ni^2+^ measurement. The coating layers of the Ni^2+^-imprinted CS gel were optimized by refractive index sensing experiments. Ni^2+^ solutions with different concentrations were prepared to investigate the sensing performance, whereas copper ions (Cu^2+^), sodium ions (Na^+^), and potassium ions (K^+^) were used to compare the specific measurement.

## 2. Materials and Methods

### 2.1. Materials

The materials used in our experiments were: Hydrogen peroxide solution (H_2_O_2_, 37%), sulfuric acid (H_2_SO_4_, 95% Laboratory Reagent Grade), gold chloride hydrate (HAuCl_4_.3H_2_O), sodium citrate tribasic dehydrate (Na_3_C_6_H_5_O_7_.H_2_O), medium molecular weight chitosan, 190–310 kDa (MCh), polydimethyl diallyl ammonium chloride (PDDA), epichlorohydrin (ECH), nickel chloride hexahydrate (NiCl.6H_2_O), ethylene diamine tetra-acetic acid (EDTA), sodium chloride (NaCl), potassium chloride (KCl), and copper chloride (CuCl_2_).

### 2.2. Synthesis Method of the AuNPs

The preparation method for the AuNPs in our experiments was based on a reduction of chloroauric acid with sodium citrate [28]. First, 100 mL of chloroauric acid solution (0.3 mM) was boiled in an oil bath; then, 10 g of sodium citrate solution (1 wt%) was mixed with it and stirred to fully react. Five minutes later, the mixed solution turned wine red, indicating that AuNPs were gradually being produced. After 30 min of reaction time, the mixture was removed from the oil bath and was continuously stirred until it cooled to room temperature. Finally, the AuNPs solution was stored in a refrigerator at 4 °C.

### 2.3. Preprocessing of the Optical Fiber

The sensing fiber used in the experiment is a hard polymer cladding multimode optical fiber (HP2140-A from YOFC), which has a three-layer step index profile with a core diameter of 200 μm, a cladding diameter of 230 μm, and a coating diameter of 500 μm. The materials of the three layers are pure silica glass, fluoroacrylate, and ethylene tetrafluoroethylene plastic, respectively. The sensing fiber has a high numerical aperture of 0.37, which enables good optical coupling and optical receiving. A 2-cm long coating of the optical fiber in the middle section was stripped mechanically, and then the middle section was immersed in an acetone solution to remove the hard polymer cladding.

### 2.4. Functionalization of the Optical Fiber

**Figure 1:**
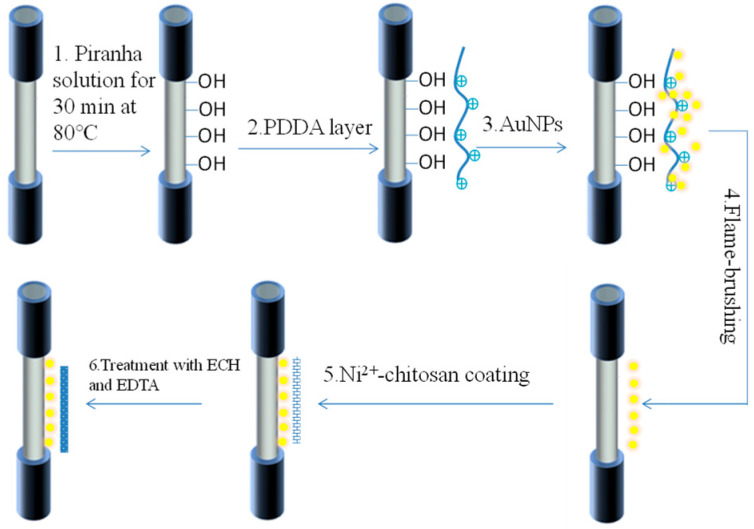
Functionalization of the proposed LSPR sensor based on Ni^2+^-imprinted chitosan.

Figure 1 shows the functionalization of the optical fiber. The ion-imprinted chitosan-based LSPR optical fiber sensor was prepared in three main steps. The first step is modifying the optical fiber with AuNPs, called Au-OF. The second step is coating the Au-OF with Ni^2+^–chitosan sol., called Ni^2+^-CS-OF. The final step is ion imprinting, called Ni^2+^-imp-CS-OF.

The middle bare section of the multimode fiber was immersed in piranha solution for hydroxylation of the core surface. A PDDA nanosheet was coated on the core surface after immersing the fiber in PDDA solution (30 wt%) for eight hours. The AuNPs were immobilized on the PDDA nanosheet with electrostatic interaction, and the unbounded AuNPs were removed by rinsing the fiber with ultrapure water. Then, the optical fiber was put into an oven for 20 min at 60 °C. The middle section was annealed at a high temperature with a flame brush. The flame brush technology cleaned the surface of the AuNPs by burning out the organic stabilizers and linkers, enabled the AuNPs to merge, and improved the agglomeration phenomenon. The process ensured that AuNPs were evenly dispersed on the optical fiber substrate and that Au–OF was obtained.

To obtain the Ni^2+^–chitosan sol., 0.08 g chitosan was first uniformly dispersed in 10 mL acetic acid solution by stirring for 12 h. After stirring, the chitosan sol. was clear and transparent, and the small bubbles in it almost disappeared. Then, 0.01 g NiCl_2_ was dissolved into the chitosan sol. by stirring for 2 h. Several layers of Ni^2+^–chitosan sol. were coated on the Au-OF using the dip coating technique. The top section of the optical fiber was fixed on the dip coater, and the middle section was immersed in the Ni^2+^–chitosan sol. After several trials, a uniform layer was successfully obtained by withdrawing the fiber at a speed of 500 mm/min. The coating layer was dried and curved for 2 h at 60 °C. Finally, the Ni^2+^-CS-OF was completed by coating the Ni^2+^–chitosan sol. on the Au-OF. Six samples with different coating layer thicknesses were obtained by repeating the dip coating process.

In the ion imprinting process, the Ni^2+^-CS-OF was first immersed into the ECH for 2 h to strengthen the cross-linking degree between Ni^2+^ and chitosan. The sensor was soaked in the EDTA solution to release the Ni^2+^ ions, and then countless voids were formed in the chitosan molecule. Finally, the LSPR optical fiber sensor with Ni^2+^ ion-imprinted chitosan was completely ready for Ni^2+^ sensing, and Ni^2+^-imp-CS-OF was obtained.

### 2.5. Experimental Setup

**Figure 2:**
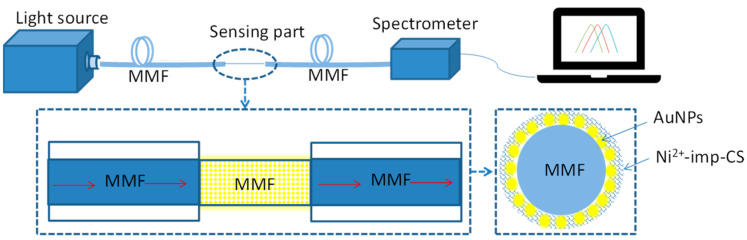
Experimental setup of the proposed Ni^2+^ sensor based on LSPR. MMF: Multi-mode fiber; AuNPs: Au nanoparticles; Ni^2+^-imp-CS: Ni^2+^ imprinted chitosan.

Figure 2 shows the experimental setup for measuring the refractive index and the heavy metal. Both ends of the sensing fiber were grinned with abrasive papers and then terminated with two fiber jumpers using the fiber adapters and FC-SMA connectors. One jumper was connected to a halogen light source (HL1000, wyoptics in Shanghai, China) and the other one was connected to a spectrometer (USB 4000, Ocean Optics in Florida, US). A basis spectrum was first recorded by connecting a bare fiber instead of the sensor fiber, and then the absorption spectrum was monitored under different solutions for an average of 10 times.

## 3. Results

### 3.1. Characterization of the Sensor Device

**Figure 3:**
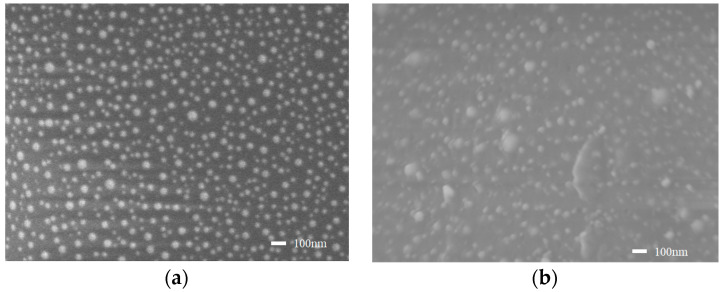
SEM micrographs of (**a**) Au-nanoparticle-coated optical fiber and (**b**) four layers of chitosan-gel-coated optical fiber.

**Figure 4:**
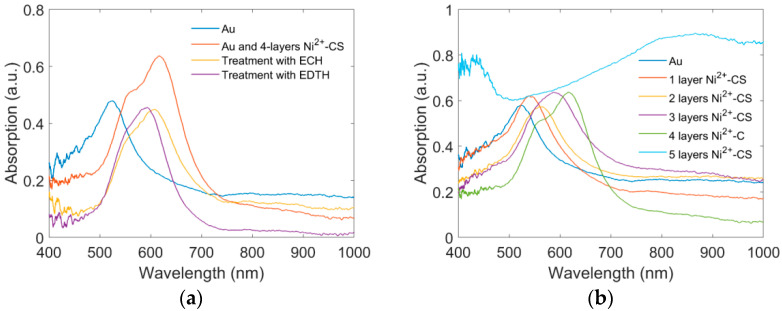
Absorption spectra of the LSPR sensor (**a**) in the preparing process and (**b**) with different layer chitosan gels.

Figure 3a shows the morphology of the gold nanoparticles on the optical fiber. It is found that the size, shape, and distribution of the gold nanoparticles were relatively uniform. Figure 3b shows the surface morphology of Ni^2+^-CS-OF. The transparent film in the picture suggests that the chitosan gel was successfully modified on the surface of the gold nanoparticles. Figure 4a shows the absorption spectra in the sensor preparation process. The absorption spectrum of the Au-OF has a relatively narrow absorption band, and the absorption peak is located at 523 nm. After coating with four layers of Ni^2+^–chitosan sol., the refractive index of the surrounding environment was increased, and hence the absorption peak appeared as a red shift. Treatment with ECH created a slight blue shift in the absorption spectrum. After that, the Ni^2+^-CS-OF was treated with the EDTA eluent, and the Ni^2+^ embedded in chitosan molecules were removed, forming numerous Ni^2+^ ion voids in the coating material. With the desorption of Ni^2+^, the cross-linkage between Ni^2+^ and chitosan is released, and the refractive index of the coating material decreases. Therefore, the absorption band illustrates a blue shift. The final absorption peak wavelength of the LSPR sensor was still larger than that of the Au-OF.

We also assessed the influence of the chitosan’s thickness on the absorption spectra by preparing six samples with different layers of Ni^2+^–CS gel. Different Ni^2+^–CS gel layers were coated on Au-OF using the dip coating method, as shown in Figure 4b. With the increase in the number of layers, the refractive index around the optical fiber gradually increases, and the position of the absorption peak continues to produce a red shift. The absorption peaks are located at 523, 542, and 563 nm for the sensors with zero, one, and two layers of Ni^2+^–chitosan gel, respectively. Even though the absorption band occurs as a red shift when increasing the coating thickness, only one absorption peak was observed in the whole spectrum, corresponding to the transversal LSPR resonance peak. For the third and fourth layers of the Ni^2+^–CS gel, the transversal peak still existed, but a longitudinal peak was dominant in the spectrum. The appearance of longitudinal peak originates from the agglomeration of AuNPs. The absorption spectrum of the five layers of Ni^2+^–CS gel was so degraded that no obvious absorption peak could be found.

**Figure 5:**
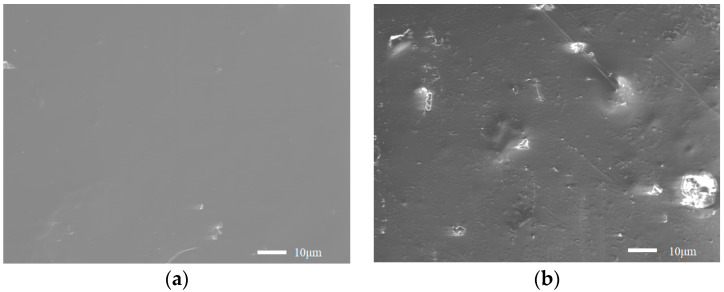
SEM micrographs of (**a**) the Ni^2+^–chitosan film and (**b**) the film after EDTA treatment.

Figure 5 shows the SEM micrographs of the Ni^2+^–chitosan film and the film after EDTA treatment. It is found that the Ni^2+^–chitosan film had a smooth and flat surface, but the surface of the film after EDTA treatment was much rougher. The EDTA destroyed the chelation of the Ni^2+^ ions by the amino and hydroxy groups of chitosan, and the release of the Ni^2+^ ions and production of the voids made the film rough.

### 3.2. Refractive Index Response

**Figure 6:**
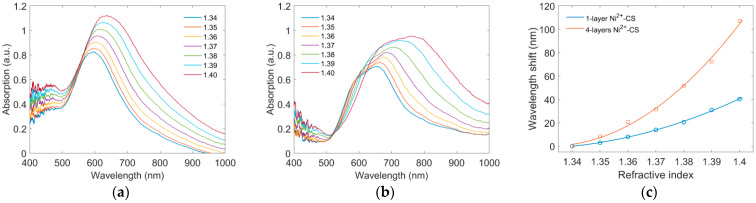
Refractive index sensing of the LSPR sensor with difference thickness of the Ni^2+^–CS gel. Absorption spectra of the LSPR sensor with (**a**) one layer of Ni^2+^–CS gel and (**b**) four layers of Ni^2+^–CS gel. (**c**) Comparison of the wavelength shift.

Before Ni^2+^ ion sensing, the refractive index sensitivity of the optical fiber sensors with different coating thicknesses was tested in sucrose solutions with different concentrations. Figure 6a,b show the absorption spectra of the sensors with one layer and four layers of the Ni^2+^–CS coating. The refractive index of the sucrose solutions varies from 1.34 to 1.40. It is found that the absorption peaks in both sensors shifted to the longer wavelength with the increase in the refractive index. Figure 6c compares the sensitivity of the two sensors. It is found that the fourlayer Ni^2+^–CS sensor had a total wavelength shift of around 110 nm, which is around two times larger than that of the one-layer Ni^2+^–CS sensor. According to the analysis in Figure 5b, the four-layer Ni^2+^–CS sensor has a longitudinal resonance peak, whereas the one-layer Ni^2+^–CS sensor only exhibits a transversal resonance peak. The longitudinal resonance peak is usually much more sensitive to the refractive index than the transversal resonance peak [28,29]. Therefore, the four-layer Ni^2+^–CS sensor was used for the Ni^2+^ ion sensing layer.

### 3.3. Ni^2+^ Detection

In Ni^2+^ solution detection, the LSPR sensor has innumerable voids made by the ion imprinting method in chitosan. The voids match the template ions in terms of size and structure. These specific imprinting sites have strong specific recognition of the target ions. Here, Ni^2+^ sensing is completed by the chelation between the Ni^2+^ ions and the chitosan. In detail, the hydroxyl and amino groups around the voids have a cross-linking force, causing the Ni^2+^ ions in the surrounding solution to become embedded in the voids again. This reaction enhances the cross-linking degree between Ni^2+^ and chitosan, thus increasing the refractive index of the entire sensing material and eventually leading to the spectral shift in the LSPR band. When the sensor is immersed in the EDTA eluent again, Ni^2+^ is eluted out due to the stronger chelation between the Ni^2+^ and EDTA, once again forming voids in the chitosan. The refractive index of the coating material also decreases with the release of the cross-linking between Ni^2+^ and chitosan, making the LSPR band shift back to the original position. Based on this theory, the sensor can be reused for testing Ni^2+^ ions with different concentrations.

**Figure 7:**
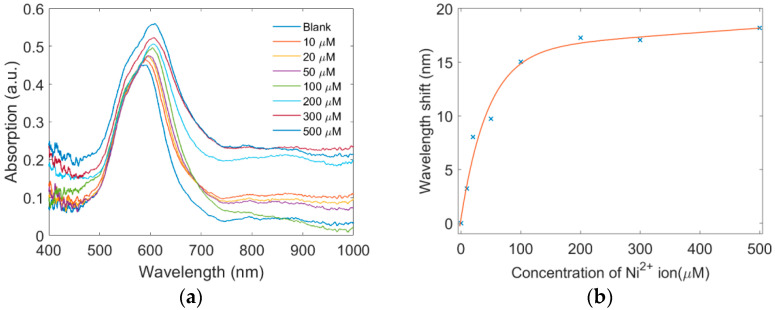
Ni^2+^ sensing based on the proposed LSPR sensor. (**a**) Absorption spectra with different concentrations of Ni^2+^ ions. (**b**) Wavelength shift as a function of the concentration of Ni^2+^ ions.

Before Ni^2+^ detection, the sensor was soaked in the blank solution for 10 min, then taken out and baked in the oven at 60 °C for 10 min. The LSPR spectrum of the blank solution was obtained. Figure 7a shows the LSPR band spectra of the blank solution and the solutions with different Ni^2+^ concentrations. It can be seen that the LSPR band shows an obvious shift. The absorption peak wavelength shifts by 10 nm when the Ni^2+^ concentration is increased from blank to 50 μM. The linear fitting of the band response indicates the proposed senor has a sensitivity of around 185 pm/μM. The limit of detection (LOD) was calculated to be 0.512 μM [30]. When the concentrate is increased to 100 μM, the sensitivity began to decrease, and the wavelength shift seems to be saturated.

The proposed sensor has a comparable LOD with the photonic-crystal-fiber-based Mach–Zehnder interferometric sensor, which was also fabricated using ion-imprinted chitosan [26]. However, the LSPR sensor enhanced the interaction between the optical evanescent field and the surrounding medium by using the nanoparticles. Hence, the proposed sensor holds higher sensitivity than that of the interferometric sensor [26,31], which have a sensitivity of 63.2 pm/μM and 55.3 pm/μM based on the photonic crystal fiber and no-core fiber, respectively.

**Figure 8:**
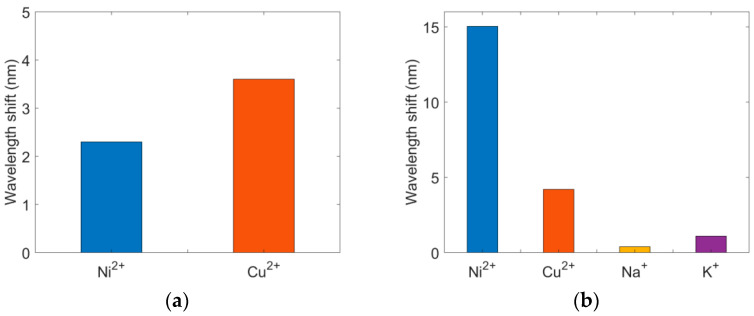
Selectivity of the LSPR sensor with (**a**) CS gel and (**b**) Ni^2+^–CS gel when the ion concentration is 100 μM.

To verify that the ion imprinting method can improve the selectivity of the sensor, we compared the sensing performance of the LSPR sensor with CS gel and Ni^2+^-imp–CS gel. In the selectivity experiments, all ion concentrations were fixed at 100 μM. Figure 8a shows the wavelength shift of the LSPR sensor with CS gel. It is found that the wavelength shifts for Cu^2+^ and Ni^2+^ are comparable. The poor selectivity is derived from the electrostatic attraction of CS. In contrast, the LSPR sensor with Ni^2+^-imp–CS gel has good selectivity for Ni^2+^ ions, as shown in Figure 8b. The special void in the Ni^2+^-imp–CS gel is ready to absorb the Ni^2+^ ion. The comparison experiments proved that the ion imprinting technology can improve the selectivity of the sensor.

**Figure 9:**
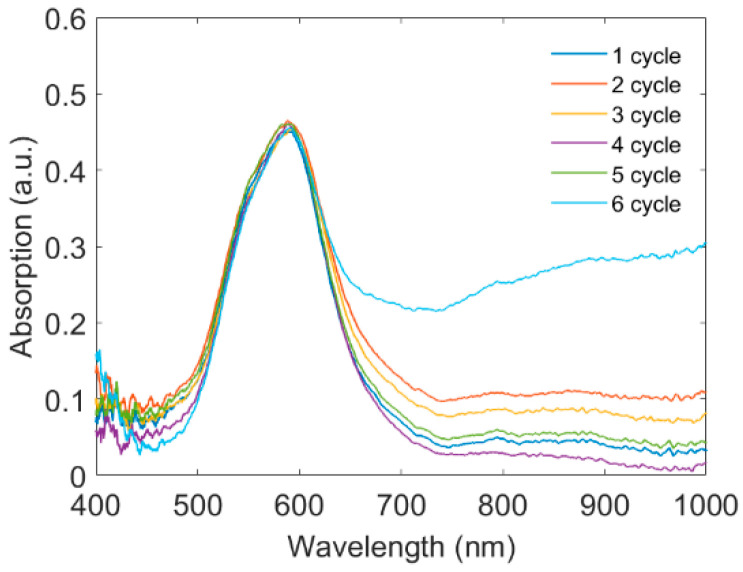
Absorption spectra of the LSPR sensor when an EDTA solution is used for releasing Ni^2+^ ions for six cycles.

The repeatability of our proposed sensor was finally investigated. Here, an EDTA solution was used as an eluent solution to expel the Ni^2+^ ions out of the CS gel to be reused in another uptake process. We repeated the eluent process six times and monitored the absorption spectra of the LSPR sensor, as shown in Figure 9. The spectra overlap, indicating the good repeatability of the proposed sensor. Moreover, the sorbent displayed considerable mechanical stability after all the performed removal–desorption cycles, and no obvious degradation or cracks were observed.

## 4. Conclusions

A Ni^2+^ detection method was proposed by using an LSPR sensor with Ni^2+^-imprinted chitosan. The LSPR was excited by coating Au nanoparticles on the multimode fiber. Ni^2+^–CS gel was successfully modified on the AuNPs using the dip coating method. It was found that four layers of Ni^2+^–CS gel can excite the LSPR’s longitudinal resonance band, which effectively improved the sensitivity of the sensor. First, an ECH was used as the crosslinking agent to fix the three-dimensional structure of the Ni^2+^ ions and chitosan gel. Then, an EDTA was used to release the Ni^2+^ ions and hence form countless voids. Finally, a Ni^2+^-imp–CS gel was prepared by using the ion-imprinting technology. In the Ni^2+^ sensing, Ni^2+^ is refilled into the voids to increase the refractive index of the sensing material, thus producing the wavelength shift in the LSPR peak and realizing the measurement of Ni^2+^. The sensitivity of the sensor to Ni^2+^ reaches 185 pm/μM, and the LOD reaches 0.512 μM. The Ni^2+^-imp–CS provides strong specific recognition of the Ni^2+^ ions in terms of size and structure. Compared with the pure CS gel, the selectivity of the sensor was largely improved. The proposed heavy metal sensor based on ion-imprinted chitosan has potential applications in environmental monitoring.

## Data Availability

Not applicable.
